# Discussing systemic racism and racial privilege at a large, academic health center using a modified privilege walk

**DOI:** 10.1186/s12909-024-05302-8

**Published:** 2024-03-22

**Authors:** Elizabeth A. Brown, Rosemarie Jones

**Affiliations:** 1 School of Community & Environmental Health, College of Health Sciences, Old Dominion University, Norfolk, VA 23529 USA; 2 Bloomberg School of Public Health, Johns Hopkins University, 615 N. Wolfe Street, Baltimore, MD 21205 USA

**Keywords:** Systemic racism, Academic medical centers, Health Equity, Race/Ethnicity, Privilege, Racial privilege, Social determinants of health

## Abstract

**Background:**

There is a motivation for organizations to understand race and racism from the perspective of minoritized individuals. Academic health centers (AHC) are ideal organizations to have these conversations as they educate healthcare providers, support research in health disparities, and care for diverse patients.

**Methods:**

We piloted and evaluated a virtual Modified Privilege Walk (MPW) with faculty, staff, and students at an AHC in July 2020 to promote difficult conversations about race/racism, social class, and privilege. Each MPW session was voluntary, held virtually over Zoom, and lasted one hour and thirty minutes. Before attending, participants answered questions based on their race/ethnicity and social class to calculate a “privilege score.” After each session, attendees were asked to complete an evaluation survey.

**Results:**

There were five virtual MPWs with 132 attendees, and 74 participants completed an evaluation survey (56% response rate). Many respondents were students (*n* = 29, 39.2%). Most respondents either agreed (*n* = 36, 48.6%) or strongly agreed (*n* = 32, 43.2%) that the virtual MPW positively impacted how they will interact with those of a different race/ethnicity. Attendees requested having more virtual MPWs with leadership, incorporating virtual MPWs in various program curricula, and requiring new employees to participate.

**Conclusions:**

American organizations, particularly AHCs, should provide safe spaces and support these discussions surrounding race and racism as many were founded, built, or operated during a time of free labor and segregation that exerted power and control over minoritized individuals. Authors provide recommendations to dismantle organizational racism and support minoritized employees, patients, and students.

## Background

As with most individuals and businesses across the globe, academic health centers (AHCs), also called academic medical centers (AMCs), have felt the effects of coronavirus disease (COVID-19) in some way or another. AHCs and AMCs serve as teaching hospitals where patients receive care and healthcare students in medicine, nursing, and some allied health professions like dentistry learn about their respective professions and how to treat patients, address health equity, and improve quality of life. Further, these healthcare institutions are charged with addressing health equity through various research methods [1]. In the past, medicine and healthcare delivery focused on the individual; however, there is a transformation occurring where healthcare institutions are focusing on improving population health by acknowledging and addressing system-level changes [2]. Education surrounding healthcare delivery for both providers and providers in training should encompasses various structural and intermediary social determinants of health (SDOH) beyond the individual (e.g., social policies, healthcare access, culture and societal values, and material circumstances [3–5]. Healthcare students attending AHCs are not only learning about various SDOH and their impact [6–10], including privilege as a SDOH [11, 12], healthcare students and those in academia are discussing approaches to work with social communities to dismantle these upstream barriers to improve health equity.

At one AHC, COVID-19 and its effects overwhelmed conversations; however, employees realized we were missing critical discussions about race/ethnicity, racism, and privilege, especially after Mr. George Floyd’s death. It was not that colleagues lacked care and concern for each other. Conversations about systemic racism and its effects were not easy to have, like the discussions about COVID-19 and its consequences. Colleagues were ill-equipped to have difficult conversations about racism and its impact on our colleagues, specifically minoritized colleagues. As we learned more details related to Mr. Ahmaud Arbrey and Ms. Breonna Taylor’s unjust murders, many of us were uncomfortable and unsure of what to say. Many colleagues did not know how to reach out to their minoritized colleagues, particularly Black Americans, who may have been struggling with the continued senseless killings of Blacks in America in addition to the harmful effects of COVID-19.

Now, there is a motivation for organizations and society to better understand psychosocial factors (e.g., racism and stress) from the perspective of minoritized groups. This is especially the case as racism relates to public health [13, 14]. Further, this motivation is especially true for AHCs who care for some of society’s most vulnerable populations. COVID-19 and the 2020 summer of racial unrest provided an opportunity to have discussions and listen to those who may be considered underprivileged and learn from their experiences. In July 2020, one college, which is home to a large AHC, decided to take a different approach and tackle these difficult conversations head-on.

Before healthcare professionals can make changes, we must first acknowledge various privileges [15, 16] and even leadership pitfalls that hinder health equity efforts [17]. Privilege may come in the form of being White (vs. racial minoritized individual), heterosexual (vs. lesbian, gay, bisexual, or transgender), wealthy (vs. impoverished), or insured (vs. uninsured). Privilege may even be generational where family members pass down opportunities to younger generations (knowledge, resources, education, income, etc.). For example, a wealthy family of professionals with higher educational status can provide monetary support and knowledge to younger generations navigating undergraduate or graduate education. Various privileges may provide immunity or protection, making it difficult to understand how some factors may negatively impact others. These often-invisible systemic forces become norms, patterns, and structures in society that can work for or against groups of people and are unrelated to their individual merit or behaviors.

These systemic and social systems can be viewed as a coin and give unearned advantages and disadvantages according to a person’s relationship with an individual system of inequality regardless of whether the individual is aware or not [18]. In Nixon’s coin model, at the bottom of the coin, those populations that are high-risk, vulnerable, marginalized, disadvantaged, or hard-to-reach, are referred to as oppressed due to the negative health effects resulting from this unfair disadvantage [18]. On the other side of same societal structures or top of the coin, individuals are referred to as privileged since they receive benefits from these same structures that they did not earn, only because they are in alignment with these structures perceived as the norm [18]. AHCs cannot afford to ignore privilege, specifically as it relates to race and racism and its impact on workers and patients [15]. The first step is being aware of privileges and then taking action to correct assumptions, thinking, decisions, and policies that do not support minoritized individuals and communities.

### Study objective

Modified Privilege Walk (MPW) sessions served several purposes: (a) provide individuals a safe space to discuss privilege, race, and racism and their impact on daily life and (b) examine if a virtual the MPW session would impact how participants view privilege and interact with people of a different race.

## Methods

### Study design

This study was an evaluation of a pilot project in which we assessed if a MPW would impact how participants view privilege and interact with people of a different race.

### Institutional review board

The Institutional Review Board (IRB) at the Medical University of South Carolina determined that this study was quality improvement (QI)/program evaluation and not human subjects research. Thus, the IRB deemed the project was not subject to further review. Data were anonymized before use. No names, session numbers, or session dates are included.

### Participants

In June 2020, one author [EAB] explained the MPW to college leadership, faculty, and staff during a regularly scheduled faculty and staff virtual meeting. Researchers may use the MPW to teach healthcare students about social determinants of health (SDOH), specifically race, racism, and social class. The MPW activity could be used to (a) learn about racial/ethnic inequities through the lens of SDOH [12, 19] and (b) promote difficult discussions surrounding race/ethnicity, racism, and privilege. Generally, groups may conduct a Privilege Walk activity with people standing in a line or holding hands [20, 21]; however, this approach was not feasible with COVID-19. In July 2020, the first author utilized email to recruit participants—faculty, staff, and graduate students—from a large AHC in the southeastern United States. Participants completed a Doodle poll to select available dates/times and were not privy to who would be in the session until about one to two weeks before the MPW session. Inclusion criteria included those affiliated with a particular college in the AHC, and participation was voluntary.

### Modified privilege walk

Before attending the virtual MPW, participants voluntarily completed a questionnaire adapted from Paul Kivel’s walk exercise [22]. Questions were subjective and based on participants’ race/ethnicity and social class. For example, based on participants’ race/ethnicity, if they are offended by residential areas having the name “plantation,” one point would be deducted. If a participant grew up with more than fifty books in their home, a point would be added. After completing the questionnaire, participants calculated a total privilege score [19]. For this MPW exercise, the highest total privilege score possible was 41, while the lowest total privilege score possible was − 41.

Participants were given the following instructions with the MPW questionnaire:

To complete the form, carefully read each question and answer to the best of your ability. Your responses are based on your perception and experiences and are not necessarily right or wrong. At the end of the exercise, calculate your final score by adding all positive ones (+ 1) then subtracting all negatives ones (-1). Please note: some participants may have a negative score.

**Fig. 1:**
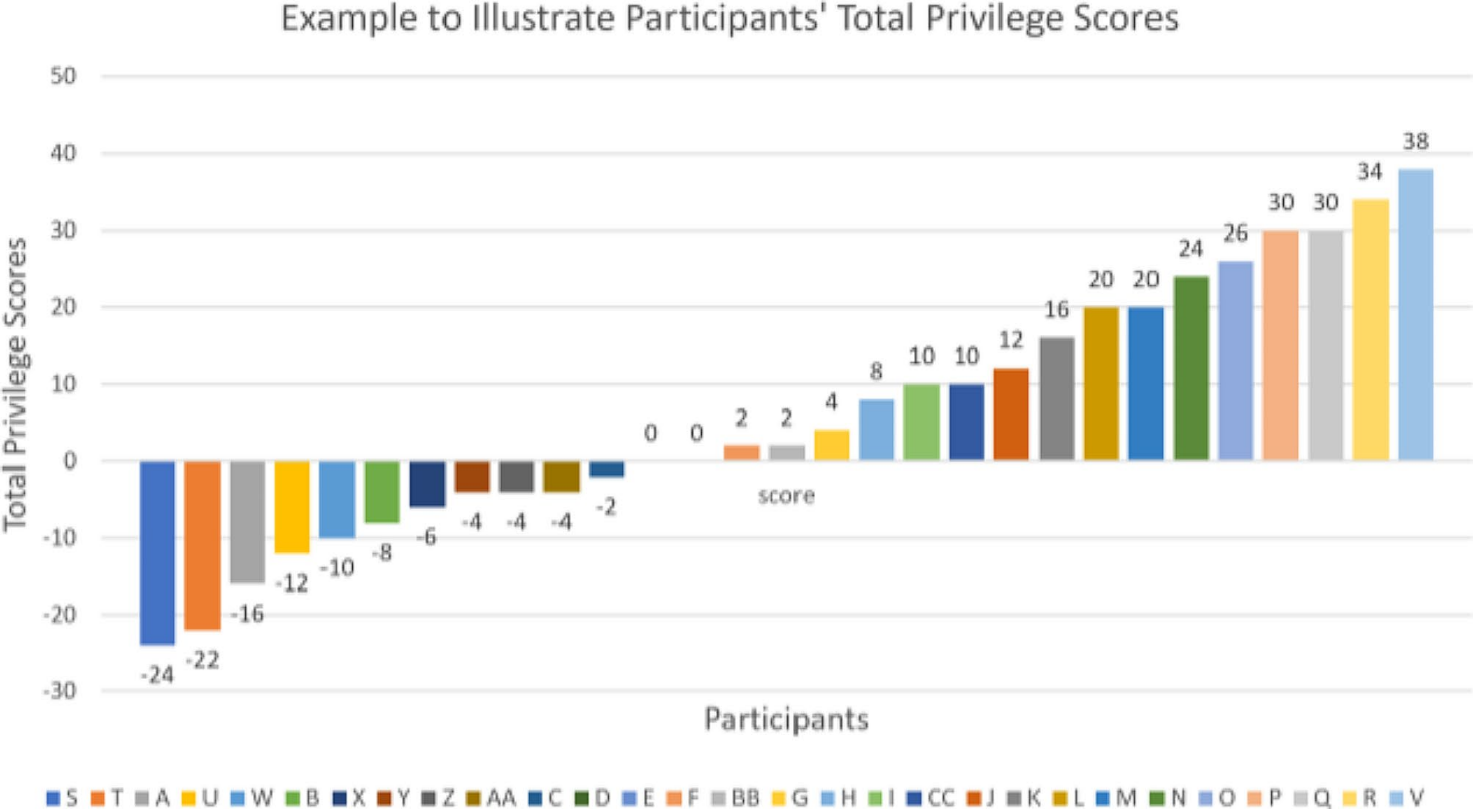
Example illustration showing range of fictious privilege scores *n* = 29. The figure contains fictitious data and does not represent any virtual MPW session. The figure illustrates how total privilege scores are shown to participants to have an in-depth conversation about privilege, race, and social class.

Then, participants sent their privilege scores to the presenter several days before the virtual MPW. The privilege scores can create a visual of privilege or opportunities based on race/ethnicity and social class, leading to challenging and provocative discussions on privilege. Figure 1 shows fictitious data to illustrate how we arrange total privilege scores for virtual MPW sessions. The presenter did not link participants’ privilege scores with participants’ names. At the bottom of Fig. 1, the letters listed are random and are not associated with any participant. Participants were encouraged to share their scores during the MPW activity only if they were comfortable doing so.

Each MPW session was held virtually over Zoom and lasted one hour and thirty minutes. Sessions begin with definitions of privilege and describe racial and health inequities related to infant mortality rates, maternal morbidity, education in primary schools, college readiness, and household debt. Participants are then given names of numerous unarmed African Americans who lost their lives to excessive violence. Names included George Floyd, Breonna Taylor, Emmett Till, Trayvon Martin, Martin Luther King, and many more. In addition to names, year of death, and activity leading up to death are discussed. At the conclusion of the lecture, attendees are shown their scores compared to others. At this time, those who have a negative score are asked to voluntarily share their stories with the group. This was done to give time and voice to those with lower social privilege scores. Doing so also ensured the stories we needed to hear were brought center stage. After we heard from those with lower scores, we opened the floor up for others to share insight and ask questions. This approach encouraged engagement from all those involved and did not require anyone share their specific score with the group unless they wanted to. To encourage participation, authors requested that those who attended a session receive 1.5 h towards the required Diversity, Equity, and Inclusion (DEI) training credit at the AHC. After each session, each attendees’ name was sent to the DEI office to ensure the participant received DEI credit for attending.

### Evaluation survey

The evaluation survey was developed, distributed, and submitted anonymously through REDCap (Research Electronic Data Capture), a secure web application designed to create and manage online surveys [23]. After completing a virtual MPW session, participants completed a voluntary evaluation survey via REDCap. Respondents described their role (faculty, staff, student, or other). We measured respondents’ level of agreement to the following statements using a Likert scale (Strongly Disagree, Disagree, Undecided, Agree, Strongly Agree):

1. The Modified Privilege Walk changed how I view privilege.
2. The Modified Privilege Walk made me more aware of my own personal privilege.
3. The Modified Privilege Walk positively impacted the way I will interact with those of a different race than myself.

Participants could also provide any additional feedback about the MPW sessions.

### Data analysis

Data were collected in REDCap and downloaded to an Excel file. We provide mean privilege scores and standard deviations (SD) for each virtual MPW. We report the number of responses using the Likert Scale for each statement listed above.

## Results

In July 2020, there were five virtual MPWs with 132 attendees. Several college and departmental leaders attended one of the sessions. After attending a virtual MPW, 74 participants completed an evaluation survey (56% response rate). Respondents were students (*n* = 29, 39.2%), staff (*n* = 27, 36.5%), or faculty (*n* = 16, 21.6%). Table 1 illustrates the average privilege scores and standard deviations for the five cohorts.

**Table 1:** Privilege scores and standard deviations (SD) from various sessions

Session^a^	Average Score (SD)
Session D	17.25 (13.19)
Session A	15.10 (13.91)
Session F	10.06 (13.40)
Session Z	16.31 (12.56)
Session J	23.17 (10.33)

^a^ Random alphabet for sessions (vs. actual session numbers) used to respect privacy

After completing the virtual MPW, survey respondents answered the following three statements:

1. The Modified Privilege Walk changed how I view privilege.
2. The Modified Privilege Walk made me more aware of my privilege.
3. The Modified Privilege Walk positively impacted the way I will interact with those of a different race.

**Fig. 2:**
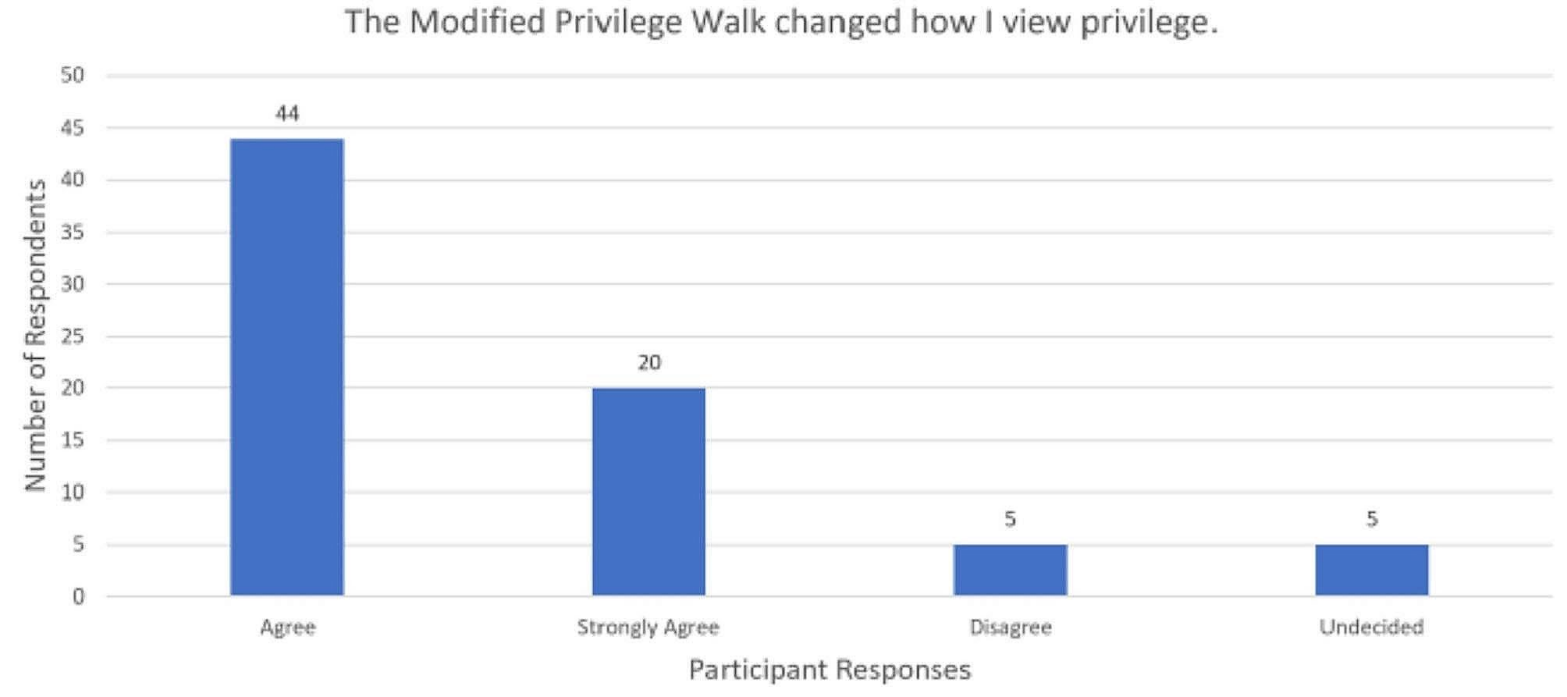
MPW and perception of privilege, *n* = 74

**Fig. 3:**
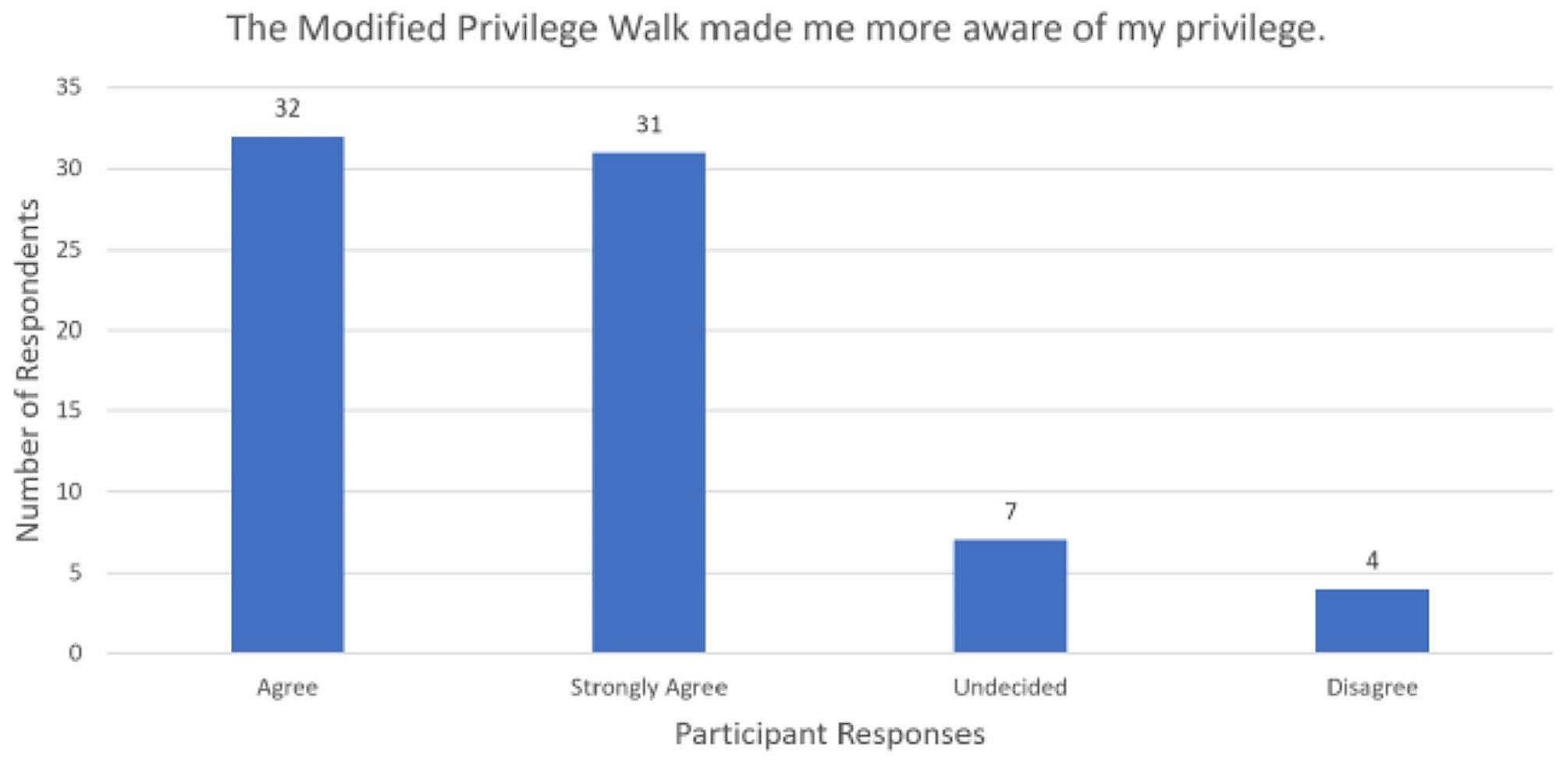
MPW and privilege awareness, *n* = 74

**Fig. 4:**
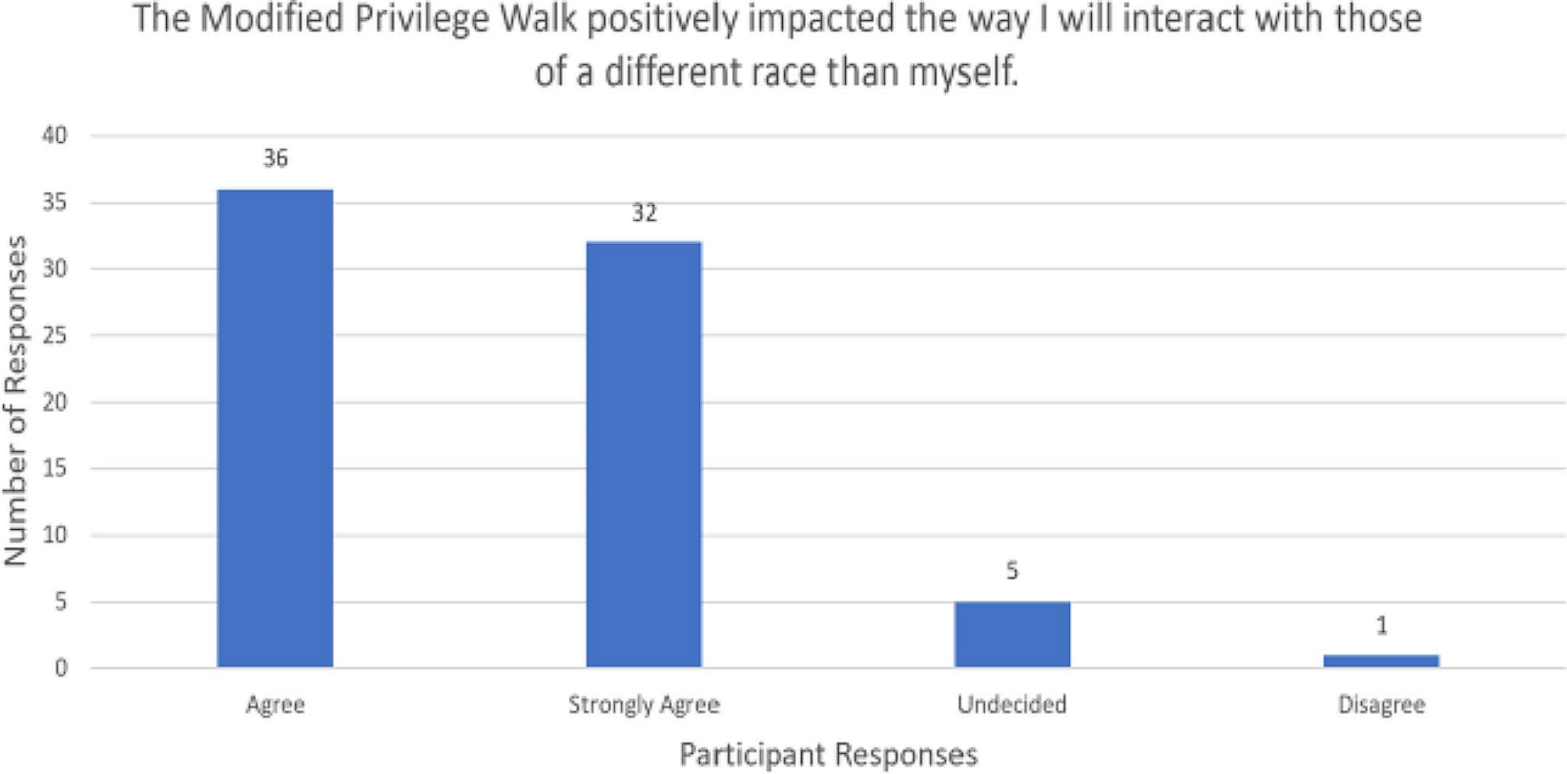
MPW and impact on interactions with people of a different race, *n* = 74

For the first statement, most respondents either agreed (*n* = 44, 59.5%) or strongly agreed (*n* = 20, 27.0%) the MPW changed how they view privilege (Fig. 2). Most participants believed the MPW made them more aware of their privilege: agree (*n* = 32, 43.2%) or strongly agree (*n* = 31, 41.9%) (Fig. 3). Many respondents either agreed (*n* = 36, 48.6%) or strongly agreed (*n* = 32, 43.2%) that the virtual MPW positively impacted the way they will interact with those of a different race (Fig. 4). None of the survey respondents selected “strongly disagree” for the three statements listed above.

Respondents provided open-ended feedback to strengthen future MPW sessions. Below are several recommendations:

1. Offer session several times a year to college leadership, faculty, staff, and students;
2. Require the session for new students and new hires;
3. Create Zoom breakout rooms with discussion prompts for more in-depth discussions with smaller groups;
4. Add MPW activity and diversity talks into academic program curricula; and
5. Continue promoting difficult discussions related to race/ethnicity, racism, social class, and privilege.

## Discussion

Over 130 attendees from the college participated in the virtual MPW, and more than half of them completed a voluntary survey. According to the survey responses, participants felt the virtual MPW impacted how they viewed privilege and made them more aware of their privilege. Many participants also agreed the virtual MPW would positively impact how they interact with people of a different race. The privilege score allowed participants to see, quantitatively, how privilege can fall on a spectrum from a negative privilege to more positive experiences with privilege.

Our college approached diversity, inclusivity, equity, and SDOH innovatively, using technology to promote difficult discussions on race, social class, and privilege in America. The first author worked to make privilege a “seen” phenomena using data and figures in Excel. Using technology to host virtual MPWs is an innovative, feasible, and efficient manner to promote difficult discussions on SDOH, systemic racism, and privilege in our society, especially when public health experts advise physical distancing to avoid COVID-19 transmission.

The virtual MPW consisted of a lecture highlighting various SDOH data in the state (e.g., maternal morbidity, infant mortality, education, employment, income, and systemic racism) and their impact on health equity. When discussing racism and its deadly effect, participants learned how COVID-19 is similar to racism and its effects [16]. To illustrate racism and its excessive and deadly consequences, the presenter shared stories of countless Black Americans who lost their lives to senseless violence for nonviolent offenses: Mary Turner, Medgar Evers, Trayvon Martin, Tamir Rice, Freddie Gray, Philando Castille, and many more. The lecture served as an opportunity to not only call attention to data demonstrating racial disparities but also the intersection of race (racism) and other SDOH, specifically education attainment, employment, income, and debt between whites and non-whites. While these are common SDOH discussed across healthcare-affiliated programs, it is plausible individuals do not consider the multiplied or compounded effect of race (racism), poverty, higher infant mortality rates, lower education, and increased debt on health and well-being.

The virtual MPWs provided an opportunity for minoritized individuals to share their emotions and struggles surrounding structural racism and classism and the impact on their lives. Structural racism is considered the historical and current policies, practices, and norms that create and support a system where Whites have better advantages in various systems, including housing, financial, judicial, etc. [24]. Classism refers to creating a preference for higher socioeconomic status (SES) or social status and looking down on those with a lower SES. Those with a lower privilege score led the discussion if they were comfortable doing so. The virtual MPW allowed attendees to participate from the comfort of their homes. We could virtually host MPWs where attendees struggled, cried, laughed, and shared their vulnerabilities without wearing a mask to cover their emotions and facial expressions. Audience members were privy to individual stories from those with less privilege to learn about the impact of race (racism) and compounded SDOH, such as poor healthcare access due to race during a global pandemic. Privileged colleagues have an opportunity to be empathetic and try to experience what those with less privilege feel. Whether it is fear walking in the neighborhood, fear of law enforcement based solely on skin color, or other worries and anxiety based on skin color and racism, those who are immune to those stresses can listen and help change things for the better. These stories are not unfamiliar and experienced by the people we work with and educate in healthcare. So, the virtual sessions emphasized these stories are from people in our divisions, departments, and colleges, not from someone in a different place or necessarily just another story on the news. These stories were close to home (e.g., people we collaborate with and respect in academia) and emphasized the multiplied effect various SDOH have on health and well-being. Again, this is the pinnacle of public health—promoting health in all communities.

The field of public health and AHCs are progressively moving towards a social ecological approach to health, recognizing the social, political, and structural determinants of health and health equity in populations rather than an individual biomedical paradigm, and yet, health practice, research, and education persists to endorse individual/interpersonal measurements and interventions leading to only more unanswered questions. To advance health equity, there needs to be an understanding of the role SDOH influence individuals’ circumstances, choices, and behaviors impacting their life and health outcomes. Many studies conclude that health disparities and outcomes do not result from only genetics and individual behaviors, but from social structures, systems, and policies. These same structures, systems, and policies are significant barriers to the work of health equity and the evolving social ecological understanding of health. This biomedical paradigm is often biased towards quantitative studies when qualitative studies provide more meaningful answers to individual community health needs. Funding structures and requirements, academia, and researchers all cling to these deeply-rooted practices only preserving the same inequities that public health is seeking to address [25]. 

In later discussions, there was a realization that American organizations, including AHCs, were founded and built during a period where minoritized individuals, particularly African Americans and Blacks, were either used as slaves for free labor or suffered under various social policies like Jim Crow laws and segregation, which were created to exert power and control resources and privileges like economic wealth, education, and voting privileges. Unfortunately, today, we still see how these laws, policies, and organizational structures impact quality of life and health outcomes [26, 27]. Because of this distinct connection between historical racial policies and the development of institutions and organizations, we should promote safe spaces to have these conversations about race, power, and privilege in the workplace. Further, what does that conversation look like for faculty in academia, staff members, healthcare students, and even patients visiting AHCs? How do we facilitate those conversations with various groups? One way to incorporate race/racism and organizational practices into conversations is asking ourselves, “Why do organizations (e.g., historically black colleges and universities or the African Methodist Episcopal Church) exist, and how does race/racism, privilege, and power come into play in that answer?” [28] One author advises that discussing organization and management policies and practices, without the lens of race and its impact, is a disservice to organizational stakeholder because race is embedded in institutions and their policies, therefore, race and racism are inherently embedded in organizations (e.g., our workplaces, school, communities, etc.) [26]. While social movements and legislation can mobilize change, we need leaders who are ready to make changes in various organizations, allocating resources to the necessary diversity programs to improve the quality of life of not only patients in AHCs but also the employees and students.

### Time for change: action items

Before several virtual MPW sessions ended, participants wanted to know what the solution was moving forward. How could they use their privilege to make changes for those who may be less privileged? Other than voting, how could they make changes to systems of oppression? We admit many changes need to come from lawmakers and policies that support better outcomes for minoritized groups in various systems, including education, healthcare, and judicial systems. However, the presenter gave participants a few recommendations to consider as “low-hanging fruit” and much more attainable at the individual and college-level versus national. It is important to note that participants may be administrators, leaders, or managers with influence concerning decision making and policies relevant to student recruitment, faculty searches, and mentorship. Their participation and engagement in these sessions is paramount to impact systems changes and increase opportunities or privileges for students, staff, and faculty who may have lower privilege scores due to compounded SDOH like race (racism), poverty, etc. For example, a hiring manager may understand how, for certain positions, requiring a college degree may create a structural barrier for someone, specifically racially-minoritized individuals or individuals with lower education attainment, who does not have a degree but has years of experience. Action items included focusing on the recruitment and retention of minoritized individuals, mentorship and sponsorship, and advocacy. For example, mentors and sponsors actively providing equitable opportunities related to training, research, and teaching support or connecting minoritized students and junior faculty with researchers and leaders in your professional network and field are just a few approaches to using privilege in a positive manner.

### Limitations

The authors did not establish survey validity or reliability. The survey did not prompt respondents to answer why they chose the following responses: “undecided,” “disagree,” or “strongly disagree.” Thus, it is unclear why respondents may have provided these responses. Participants manually completed the MPW questionnaire, which may have led to errors with their total privilege score. Now, participants complete the MPW questionnaire via a secure, electronic system where the application calculates the respondent’s privilege score, which should decrease errors with calculating total privilege score.

## Conclusion

The virtual MPW cannot assess if participants will change or act on these recommendations in the long-term. However, the activity is an engaging approach to promoting difficult discussions about privilege, race/ethnicity, social class, and racism in America. Health professionals at AHCs, including clinicians, researchers, administrators, faculty, staff, and students, may benefit from hearing stories from those who are less privileged because they may mirror many of the diverse patient populations they serve. Suppose we continue these discussions and activities to listen to stories from the historically marginalized and minoritized? Using this approach, we may be more empathetic to our patients and colleagues concerning various SDOH, such as racism, social class, and privilege. Future research should continue exploring these difficult topics with healthcare professionals and students and develop approaches to measure privilege as a social determinant of health.

## Data Availability

All figures created using Excel. The datasets generated during and/or analyzed during the current study are not publicly available as it may allow others to identify participants. For additional details concerning data analysis, please contact Dr. Elizabeth A. Brown at eabrown@odu.edu.

## Abbreviations

AHC: Academic Health Centers
AMC: Academic Medical Centers
COVID-19: Coronavirus Disease
IRB: Institutional Review Board
MPW: Modified Privilege Walk
QI: Quality Improvement
REDCap: Research Electronic Data Capture
SDOH: Social Determinants of Health
